# Millimeter wave negative refractive index metamaterial antenna array

**DOI:** 10.1038/s41598-024-67234-z

**Published:** 2024-07-11

**Authors:** Rao Shahid Aziz, Slawomir Koziel, Anna Pietrenko-Dabrowska

**Affiliations:** 1 Engineering Optimization and Modeling Center, Reykjavik University, 102 Reykjavik, Iceland; 2 Faculty of Electronics, Telecommunications and Informatics, Gdansk University of Technology, 80-233, Gdansk, Poland

**Keywords:** Engineering, Physics

## Abstract

In this paper, a novel negative refractive index metamaterial (NIM) is developed and characterized. The proposed metamaterial exhibits negative effective permittivity (*ε*_*effe*_) and negative effective permeability (*µ*_*effe*_) at millimeter wave frequency of 28 GHz. This attractive feature is utilized to enhance the gain of a microstrip patch antenna (MPA). Two thin layers of 5 × 5 subwavelength unit cell array of NIM are placed above a single MPA to enhance the gain of the antenna. Each unit cell has an area of 3.4 × 3.4 mm^2^. A gain increase of 7.9 dBi has been observed when using the proposed NIM as a superstrate. Furthermore, the NIM array is placed over a 2 × 2 array of MPAs with four ports to demonstrate versatility of the metamaterial. The total size of the 2 × 2 antenna array system with N-MTM is about 61.1 × 34 × 16mm^3^ (5.71*λ* × 3.18*λ* × 1.5*λ*, where *λ* is the free-space wavelength at 28 GHz). The measurement result indicate that the maximum gain of the antenna array is 13.5dBi. A gain enhancement of 7.55 dB in E-Plane and 7.25 dB in H-Plane at the resonant frequency of 28 GHz is obtained. The proposed antenna structure is suitable for 5G millimeter wave communications, in particular, for possible implementation in future millimeter wave access points and cellular base stations.

## Introduction

Negative metamaterials (NIMs) are artificial structures that can manipulate electromagnetic (EM) waves in ways that are beyond the capabilities of natural materials. In particular, they may exhibit negative values of effective permittivity and permeability^1^^–^^2^. As a consequence, they allow for bending EM waves in the direction opposite to that associated with normal materials^3^. NIMs have a potential to be incorporated into 5G millimeter access points and future 6G communication systems, especially for implementing reconfigurable intelligent surfaces (RIS) and terahertz antennas. RIS are thin layers of metamaterials that can reflect or refract signals to enhance the coverage and performance in urban areas, where direct line-of-sight is blocked by objects^4^^–^^5^. Also, terahertz antennas that will operate within 0.1–10 THz frequency range, expected to be used in 6G communication, will be based on metamaterials. These artificial structures help overcome the challenges related to high path loss, low power, and limited bandwidth in this frequency range^6^^–^^7^.

NIMs have demonstrated many applications in antenna design, especially in the context of beam steering, gain enhancement, wavefront shaping, and reconfigurability^8^^–^^10^. Many researchers have investigated gain enhancement using metamaterials as reported in numerous studied, e.g.,^11^^–^^19^. For example, in^12^, zero-index metamaterial is designed and implemented over a single circular polarized patch antenna. The final design incorporates a 9 × 9 array of zero-index metamaterial to enhance the antenna gain. The reported peak gain is 12.31dBic at 7.45 GHz. In^13^, a lens based on negative metamaterial is proposed to enhance the gain of the feed antenna over a range of frequencies from 25 to 31 GHz. The specific NIM design is a well-known split ring resonator (SRR) unit cell, used to design the 8 × 8 metamaterial array, which is then allocated above the slotted patch antenna to enhance its gain to 12.7dBi. Another (transmission type) NIM-based lens, proposed in^14^, is employed to enhance the gain of the antenna. The architecture includes double-stacked NIM layers placed over a patch antenna. The obtained gain enhancement is 8.55 dB in the H-plane and 6.20 dB in the E-plane, both at the resonating frequency 10 GHz. In^15^, a multi-layer metamaterial-based antenna is designed to produce high gain over wide frequency range. Two layers of metamaterial are utilized to enhance the directivity of the antenna over the C and X bands. The achieved gain improvement is about 8.5 dB at 8 GHz.

Despite these advancements, the majority of reported antenna systems involving NIMs, exhibit various limitations. Some of the proposed structures are bulky, other designs are geometrically complex and often feature multilayer implementations, therefore, they are difficult to fabricate. Also, extending single antennas to array systems is challenging, and it is difficult to tune the devices to higher frequency bands. In order to address the aforementioned issues, in this work, a novel NIM is developed and implemented for applications in millimeter wave communication systems. The unit cell of subwavelength size is imprinted on both sides of a thin layer of dielectric material. It is meticulously analyzed and shown to possess negative effective permittivity and negative effective permeability. Two layers of unit cells are extended to form a 5 × 5 array and placed above a conventional microstrip patch antenna (MPA) as a supersubstrate. Using this configuration, a significant gain enhancement of the MPA is observed. For further investigation, the NIM array is placed over a 2 × 2 array of MPAs. It is demonstrated that the NIM layer enhances the gain of the array elements by about 7.55 dB in the E-plane and by 7.25 dB in the H-plane at the resonant frequency of 28 GHz. The total size of the entire system is only 5.71*λ* × 3.18*λ* × 1.5*λ* with *λ* being the free-space wavelength. These results are indicative of a suitability of the proposed NIM for implementing low-profile multi-antenna systems. Comparisons with state-of-the-art designs corroborate competitive performance of the proposed structure with the added value of geometrical simplicity and small size. It should also be emphasized that utilization of negative-refractive-index material for enhancing conventional MPA array gain has not been previously demonstrated in the literature.

## Methods

### Theoretical model: patch antenna with supersubstrate

**Figure 1:**
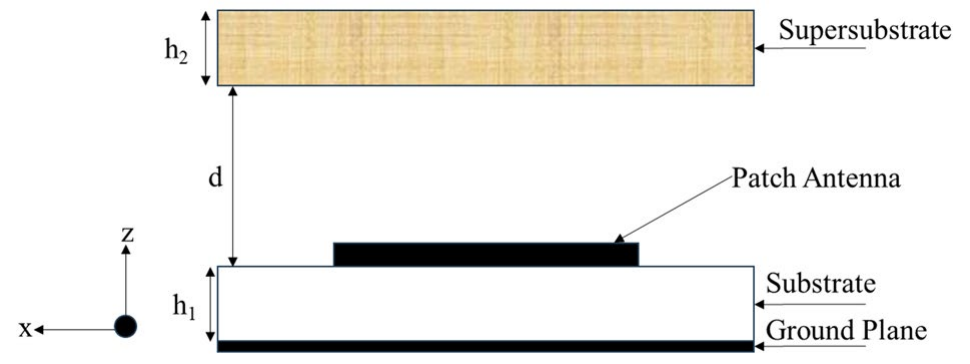
Theoretical model of a patch antenna with supersubstrate.

Figure 1 shows that a unit cell based supesubstrate is placed above a traditional patch antenna. The distance between patch antenna and supersubstrate is denoted by *d* in free space. The patch antenna is printed on a grounded substrate of thickness, *h*_*1*_, having effective permeability, *ε*_*effe1*_ and permittivity, *µ*_*effe1*_. At distance *d* from the substrate is the superstrate layer of thickness, *h*_*2*_ having effective permeability *µ*_*effe2*_ and permittivity, *ε*_*effe2*_.

To compute the far-field, there are many techniques to solve the problem. Here, first the MPA is modelled as a dielectric-loaded cavity^20^ and reciprocity theorem are applied to the whole structure (the antenna with the superstrate).

Consider E_1_, H_1_ and E_2_, H_2_ fields are generating inside the same medium with sources, J_1_, M_1_ and J_2_, M_2_, respectively. The Lorentz Reciprocity Theorem is given by,

1 $$\int {\int\limits_{S} {\left[ {E_{2} \times H_{1} - E_{1} \times H_{2} } \right]{\text{d}}S} } = \int {\int {\int_{V} {\left[ {E_{1} \cdot J_{2} + H_{2} \cdot M_{1} - E_{2} \cdot J_{1} - H_{1} \cdot M_{2} } \right]{\text{d}}V} } }$$

Here the fields (E1, H1 and E2, H2) and sources (J_1_, M_1_ and J_2_, M_2_) inside a medium is surrounded by a sphere of infinite radius. Therefore, (1) reduces to,

2 $$\int {\int {\int_{V} {[E_{1} \cdot J_{2} + H_{2} \cdot M_{1} - E_{2} \cdot J_{1} - H_{1} \cdot M_{2} ]} {\text{d}}V} } = 0$$

The patch antenna can be replaced by J1, an electric current source and M1 a magnetic current source. There are radiating an electric field, E1, H1 at point P(r, Ɵ, Φ). Similarly, a dipole with electric current J_2_ at same point P(r, Ɵ, Φ), radiates a far-field of E_2_ and H_2_. Assume M_2_ is equal to zero.

Now the far-field of the patch antenna can be derive using (2), there it is reduces to

3 $$\int {\int {\int_{V} {\left[ {E_{1} \cdot J_{2} } \right]{\text{d}}V} } } = - \int {\int {\int_{V} {\left[ {H_{2} \cdot M_{1} } \right]{\text{d}}V} } }$$

The (3) can further reduce as the reciprocity source J_2_ is,

$$J_{2} = \delta \left( {\vec{r} - \overrightarrow {{r_{p} }} } \right) \cdot \hat{u}$$

where, $\hat{u} = \hat{\theta }$ for TM—parallel polarization and $\hat{u} = \hat{\emptyset }$ for TE—perpendicular polarization.

Therefore (3) becomes,

4 $$E_{1} \left( {\vec{r}_{p} } \right) \cdot \hat{u} = - \int {\int {\int_{V} {\left[ {H_{2} \cdot M_{1} } \right]{\text{d}}V} } }$$

This equation can be used to obtain the radiation field radiating from the patch antenna to the above supersubstarte.

### Unit cell: structural design and analysis

This section introduces the geometry of the proposed unit cell. The cell’s characteristics have been studied in CST Microwave Studio using PEC and PMC boundary conditions. Additionally, the analysis of the vector surface current density has been presented and discussed.

### Unit cell model and characterization

**Figure 2:**
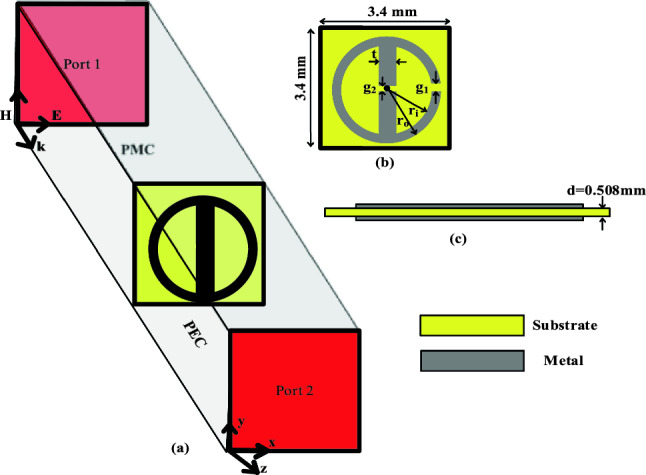
Geometry and computational model of the proposed unit cell using PMC and PEC boundary walls: (**a**) perspective view with boundary walls setup in CST, (**b**) front view, (**c**) side view.

A novel planar unit cell has been designed on a dielectric substrate for applications in millimeter-wave communication. Figure 2 shows a single element as part of a periodic arrangement, which has been implemented and evaluated in CST full-wave electromagnetic simulator^28^. To characterize the unit cell, a waveguide with perfect electric conductor (PMC) and perfect electric conductor (PEC) boundaries is employed, within which the unit cell is situated, as shown in Fig. 2a. The faces parallel to the *yz*-plane are designated as PMC boundaries, whereas those parallel to the *xz*-plane (i.e., perpendicular to the *yz*-plane), are designated as PEC. Subsequently, a linearly polarized TEM wave is introduced from waveguide port 1. This setup enables the evaluating the transmission and reflection coefficients at both Port 1 and Port 2.

Figure 2b presents the parameterized architecture of the unit cell. The cell comprises a metal ring with inner and outer radii labeled as *r*_*i*_ and *r*_*o*_, respectively, along with a gap width, *g*_1_. Furthermore, there is a strip line positioned between the ring with a thickness *t* and a gap *g*_*2*_. The entire cell topology is printed on both sides of a dielectric substrate, here, Rogers RO4003C (lossy) material, with a relative permittivity of 3.38, a tangent loss of 0.0027, and a thickness, *d* of 0.508 mm. The overall dimensions of the unit cell, including the substrate, is 3.4 × 3.4 mm^2^.

**Figure 3:**
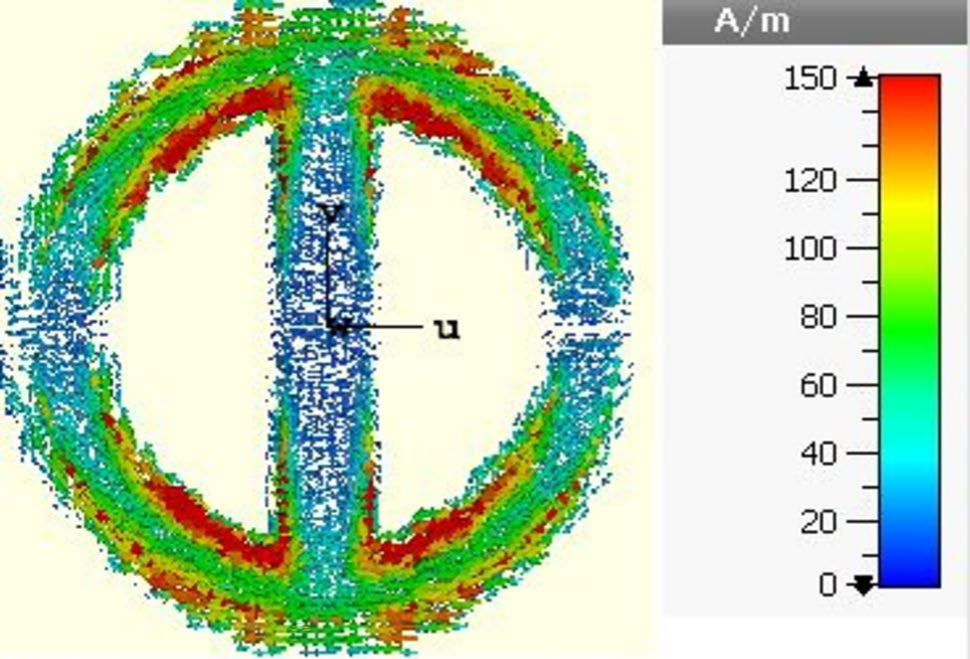
Vector surface current density calculated at 28 GHz on the top surface of the proposed element.

Figure 3 depicts a vector surface current distribution at a frequency of 28 GHz for a single unit cell. The cell is a part of an infinitely periodic arrangement of identical units. The illustration reveals strong currents aligned along the *x* and *y* directions within the upper and lower sections of the ring near the strip line junction, indicating substantial inductance effects. Conversely, in the central region of both the ring and strip, a weaker current distribution can be observed. This is indicative of a cancellation of currents within this central area. This behavior might be attributed to the introduction of a gap within the ring and strip, which induces higher capacitance effects.

**Figure 4:**
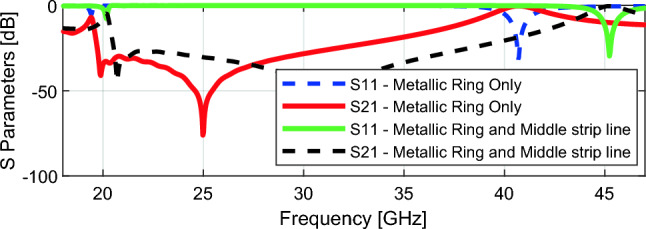
Parametric study of proposed unit cell.

Figure 4 illustrates the results of the parametric study of the unit cell. The cell characteristics are primarily determined by the metallic ring shape. The S-parameter plot indicates that the unit cell exhibits wideband transmission-type response across the millimeter-wave frequencies. With only the metal ring, the *S*_*21*_ bandwidth (at the level of –10 dB transmission) is 50% (19–38 GHz). However, the bandwidth can be further enhanced by adding a middle strip inside the ring. Consequently, the unit cell *S*_*21*_ bandwidth increases to 52% (21–43 GHz). The other two parameters, metal ring gap, *g*_*1*_ and strip line gap, *g*_*2*_ play a crucial role in fine-tuning the responses to adjust the effective permittivity, effective permeability, and refractive index, as elaborated in the next section. Table 1 shows the optimized parameters of the proposed unit cell.

**Table 1:** Unit cell designed parameters.

Parameters	Size (mm)
Metal ring outer diameter, *r*_*o*_	1.64
Metal ring inner diameter, *r*_*i*_	1.36
Metal ring gap width, *g*_*1*_	0.11
Strip line thickness, *t*	0.40
Strip line gap, *g*_*2*_	0.02

### Unit cell negative refractive index behavior

**Figure 5:**
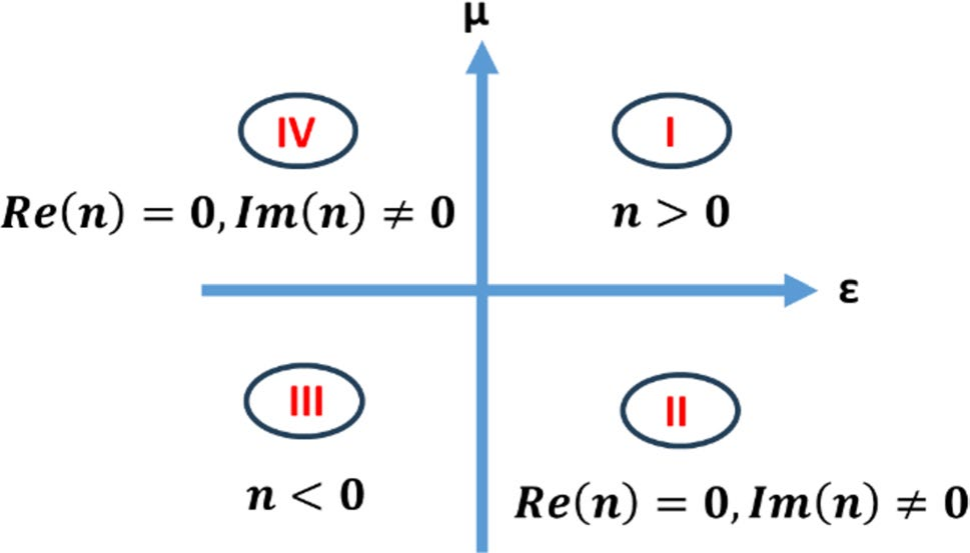
All possible combinations of *ε* and *µ*.

A material is considered to have a negative index when it exhibits both negative effective permittivity and negative effective permeability within a specific frequency range^20^. Figure 5, which shows all possible combinations of signs of *ε* and *µ*. The first quadrant corresponds to the materials with positive refraction, third quadrant corresponds to the materials with negative refraction, but the second and fourth quadrant corresponds to materials that do not permit electromagnetic wave propagation, i.e., that will simply electromagnetic waves without energy dissipation. The real part of the refractive index influences the phase velocity of electromagnetic waves propagating through the medium, affecting the phase shift experienced by the waves as they interact with the antenna structure. This phase shift can impact the constructive or destructive interference patterns of the radiated electromagnetic field, thereby influencing the overall gain of the antenna. Additionally, the imaginary part of the refractive index, representing the absorption or attenuation of electromagnetic waves within the medium, affects the propagation losses experienced by the waves as they travel through the material. Higher absorption leads to greater energy loss, reducing the overall efficiency of the antenna system and potentially limiting the achievable gain. Real part of the positive refractive index can control the propagation speed and direction of electromagnetic waves. Positive real parts of the refractive index are commonly associated with dielectric materials used in antenna design. These materials help shape the radiation pattern, impedance matching, and overall performance of antennas by controlling the electromagnetic field distribution and wave propagation within the antenna structure.

Negative index materials (NIMs) offer unique advantages in enhancing antenna performance, particularly in terms of gain. NIMs enable focusing of electromagnetic waves in the ways not achievable with positive index materials. This property allows antennas to concentrate the radiated energy in a specific direction, thereby enhancing gain in that direction. To understand the behavior of metamaterial Snell’s law is revised here,

Snell’s law describes the relationship between the angles of incidence and refraction when an electromagnetic wave passes from one medium to another with different refractive indices. It can be mathematically expressed as:

5 $$n_{1} \sin \left( {\varphi_{1} } \right) = n_{2} \sin \left( {\varphi_{2} } \right)$$

where, *n*_*1*_ = refractive index of Medium 1, *n*_*2*_ = refractive index of Medium 2, *φ*_*1*_ = angle of incidence measured from the normal to the interface, *φ*_*2*_ = angle of refraction measured from the normal to the interface.

**Figure 6:**
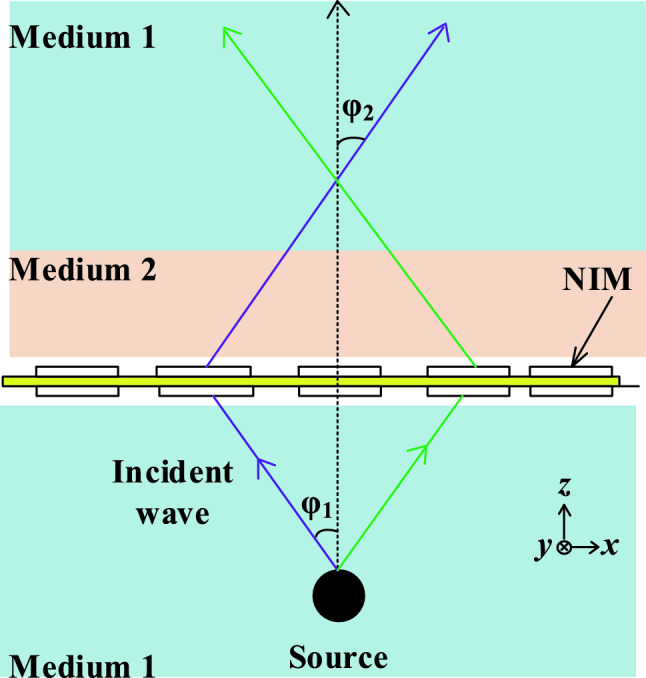
Negative refractive index effect (adapted from^21^).

Figure 6 shows the phenomena of NIMs, wherein the refraction angle (*φ*_*2*_) maintains a negative orientation with respect to the surface normal. The electromagnetic wave diverging from the source, which has an incident angle *φ*_*1*_, will converge effectively after passing through the NIMs leading to improved wave collimation ^13^. In other words, when a patch antenna impinges electromagnetic waves onto a metamaterial-based superstrate, Snell’s law dictates the relationship between the angle of incidence, *φ*_*1*_ of the electromagnetic wave and the angle of refraction, *φ*_*2*_ as it enters the superstrate. The refractive index, *n*_*2*_ of the superstrate material profoundly influences the degree to which the wave is refracted or bent. Moreover, Snell’s Law governs how the wavefronts propagate through the superstrate, impacting factors such as phase velocity and wavefront curvature.

**Figure 7:**
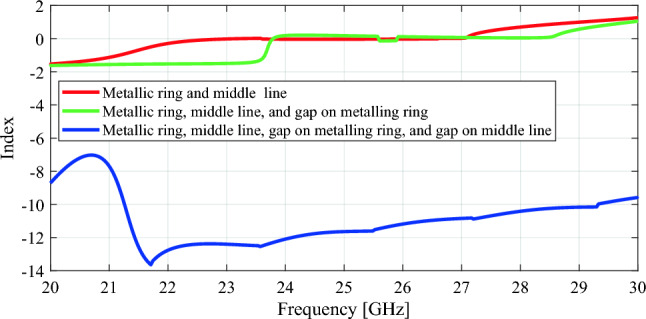
Parametric study of proposed unit cell w.r.t refractive index.

**Figure 8:**
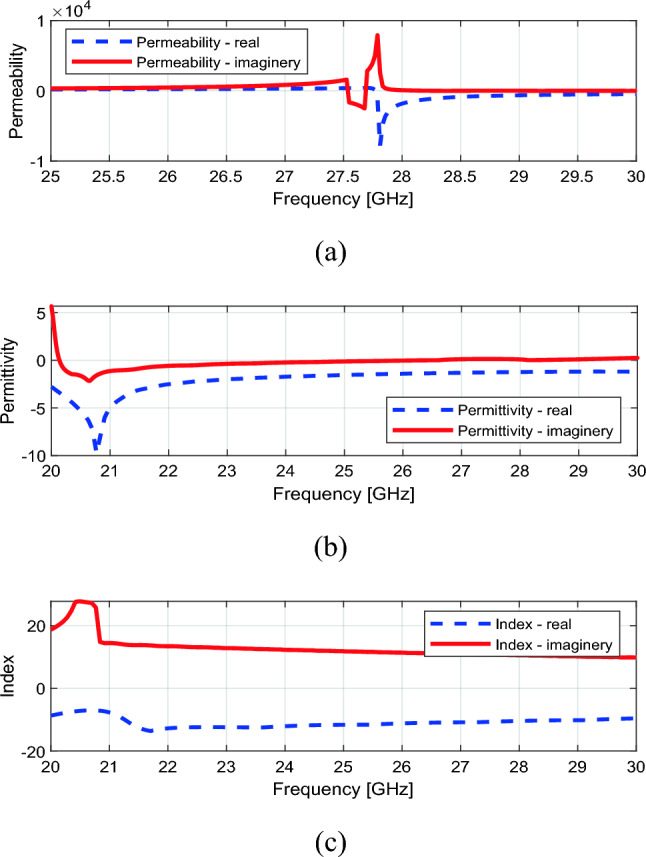
Extracted constitutive effective parameters of unit cell (**a**) Effective permittivity, (**b**) Effective permeability, (**c**) Effective refractive index.

The extracted values of refractive index *n*_*effe*_, effective permittivity *ε*_*effe*_, and effective permeability *µ*_*effe*_ of the proposed unit cell have been illustrated in Figs. 7 and 8. These parameters are computed using the following equations^22^derived from the S-parameter matrix^22^,

6 $$n_{eff} = \frac{1}{kd}\cos^{ - 1} \left[ {\frac{1}{{2S_{21} }}\left( {1 - S_{11}^{2} + S_{21}^{2} } \right)} \right]$$

7 $$Z = \sqrt {\frac{{\left( {1 + S_{11} } \right)^{2} - S_{21}^{2} }}{{\left( {1 - S_{11}^{2} } \right)^{2} - S_{21}^{2} }}}$$

8 $$\varepsilon_{eff} = \frac{{n_{eff} }}{Z}$$

9 $$\mu_{eff} = n_{eff} Z$$

where *k* and *d* are the propagation constant and material thickness, respectively. In this work, the desired operating frequency is 28 GHz.

While the parametric study of S parameters depicted in Fig. 4 illustrates that the combination of a metallic ring with a middle strip line increases bandwidth, it does not result in a negative refractive index, as can be seen by the refractive index parametric study shown in Fig. 7. At 28 GHz, the refractive index remains positive. However, Fig. 7 shows that introducing gaps between the metallic ring and the middle strip line dramatically lowers the refractive index from zero to − 10.2 at 28 GHz. This shift towards negative refractive index values offers significant advantages, particularly in enhancing the antenna gain.

In Fig. 8a, it can be observed that the effective permittivity of the proposed unit cell is negative. Similarly, the effective permeability is negative at 28 GHz. Consequently, the refractive index of the unit cell maintains a negative value at 28 GHz. This indicates that the unit cell exhibits the property of negative refractive behavior. This characteristic is expected to enhance the incident wave focusing from the source, leading to improved antenna gain.

While the metallic ring structure is common in literature, the proposed unit cell design innovatively incorporates a middle line and two gaps, enhancing unit cell functionality for wideband performance and negative refraction. Extensive parametric studies and simulations optimized factors like bandwidth, gain, and impedance matching. We view our design as a significant advancement with potential to catalyze further research and development in metamaterial-based antenna systems.

### ***Proposed unit cell as a superstrate***—***simulation results and discussion***

**Figure 9:**
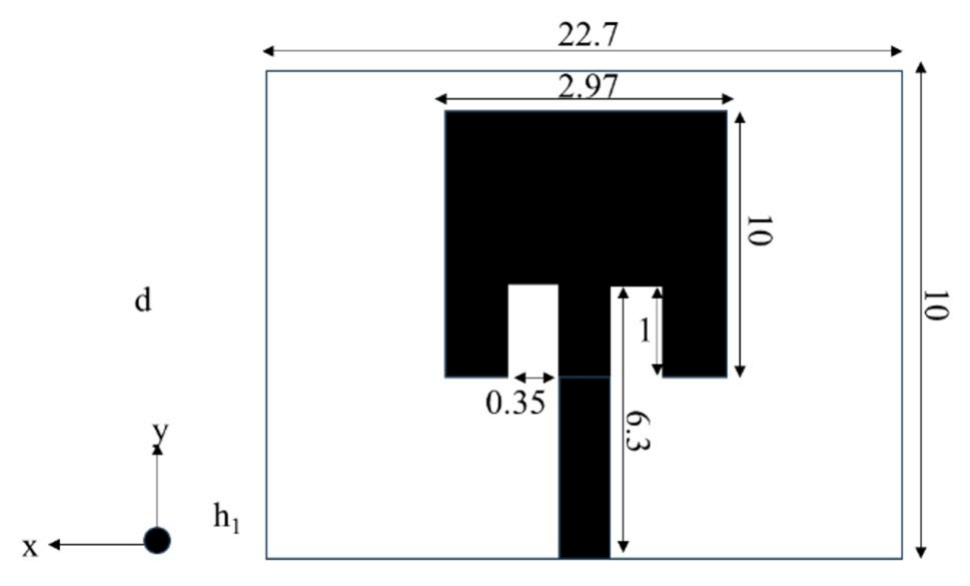
Patch antenna (all dimensions are in millimeters).

**Figure 10:**
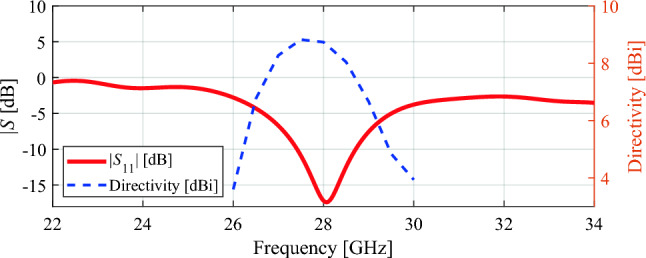
Patch antenna S-parameters and directivity.

**Figure 11:**
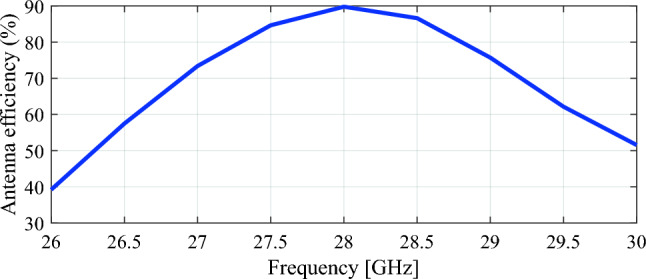
Patch antenna efficiency.

The traditional patch antenna is designed in CST software. Patch antenna parameters such as dimensions, S parameters, directivity and efficiency are shown in Figs. 9, 10, and 11 respectively.

**Figure 12:**
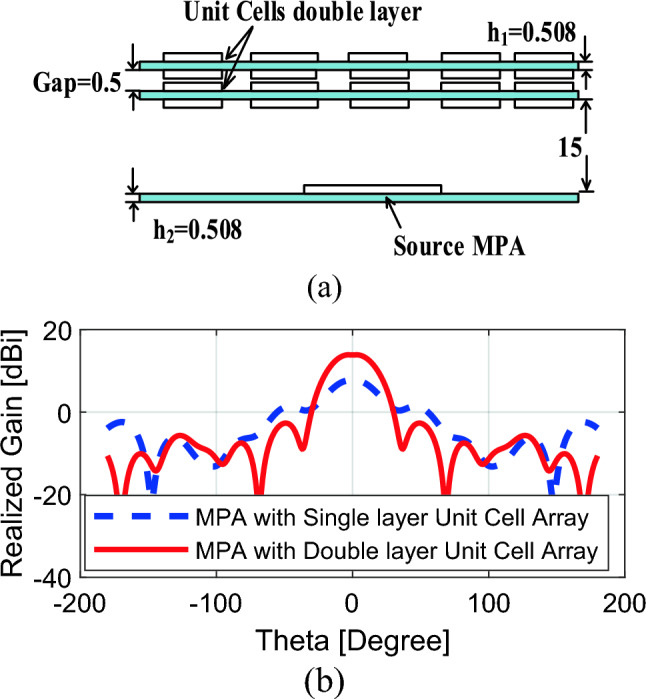
Cartesian plots of MPA with single and double layers of 5 × 5 unit cell array: (**a**) the arrangement of the two identical unit cell layers (all dimensions in millimeters); (**b**) realized gain of the MPA with a single and double unit cell arrays.

A single layer of 5 × 5 unit cell array is designed and placed on a traditional MPA. The total size of the proposed 5 × 5 unit cell array is 1.59λ × 1.59λ. The MPA is designed to resonate at 28 GHz. The optimal distance between MPA and the unit cell array is chosen as 15 mm to obtain maximum gain at 28 GHz. The obtained boresight gain of the single layer unit cell array is 7.33dBi at 28 GHz, which corresponds to about 1.33 dB gain enhancement. A careful study indicates that the boresight gain can be further increased by placing another layer of the unit cell array. The distance between two layers is 0.5 mm; however, the distance between MPA and (original) unit cell array remains the same, i.e., 15 mm. The two layers of the proposed unit cells working in synergy may lead to a significant enhancement of the gain of the source MPA. Figure 12 shows the Cartesian gain plot of the MPA with single layer and double layers of unit cell. As it can be observed, application of the two unit cell layers result in a significant gain enhancement of about 7.9dBi; the value of the improved gain is as high as 13.9dBi.

In the context of future wireless communication systems, antenna arrays stand out as promising candidates. To explore the versatility of the proposed unit cell regarding gain enhancement, a 2 × 2 MPA array incorporating the unit cell is implemented to operate at the frequency of 28 GHz. The NIM unit cell array is strategically positioned at an optimal distance of 15 mm above the 2 × 2 MPA array, resulting in gain improvement. In the following section, a of 2 × 2 MPA array with the integrated NIM unit cell is implemented and experimentally validated.

### Experimental validation

**Figure 13:**
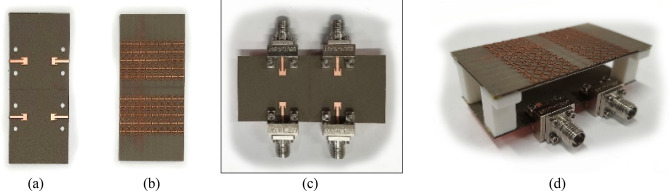
Fabricated array prototype: (**a**) 2 × 2 array, (**b**) unit cell layer, (**c**) 2 × 2 array with four ports, (**d**) perspective view of the 2 × 2 antenna array system with N-MTM.

**Figure 14:**
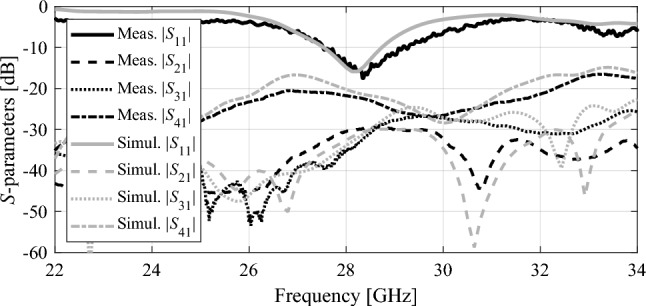
Measured and simulated S-parameters of the proposed 2 × 2 antenna array system with N-MTM at four ports.

The proposed unit cell array and 2 × 2 MPA antenna array has been fabricated on Roger’s substrate RO4003. The fabricated antenna components and 2 × 2 antenna array system with N-MTM is depicted in Fig. 13. The MPA antenna and unit cell substrates are separated by an air gap of 15 mm, which is maintained using 3D plastic spacers with relative permittivity 2.7. Likewise, the air gap of 0.5 mm between two-unit cell layers are maintained by plastic spacers. Each array element is fed individually to maintain the overall system simple and easy to integrate. Consequently, the overall system contains four ports. The *S* parameters of the proposed designs has been measured at all ports using the vector network analyzer. The simulated and measured *S* parameters are plotted in Fig. 14. During the s-parameter and radiation pattern measurements, the remaining ports were terminated by 50Ω connectors to maintain consistency and accuracy in the testing setup. The measurement result shows that MPA array elements with superstrate are resonating at 28 GHz, and isolation between them is better than 20 dB.

**Figure 15:**
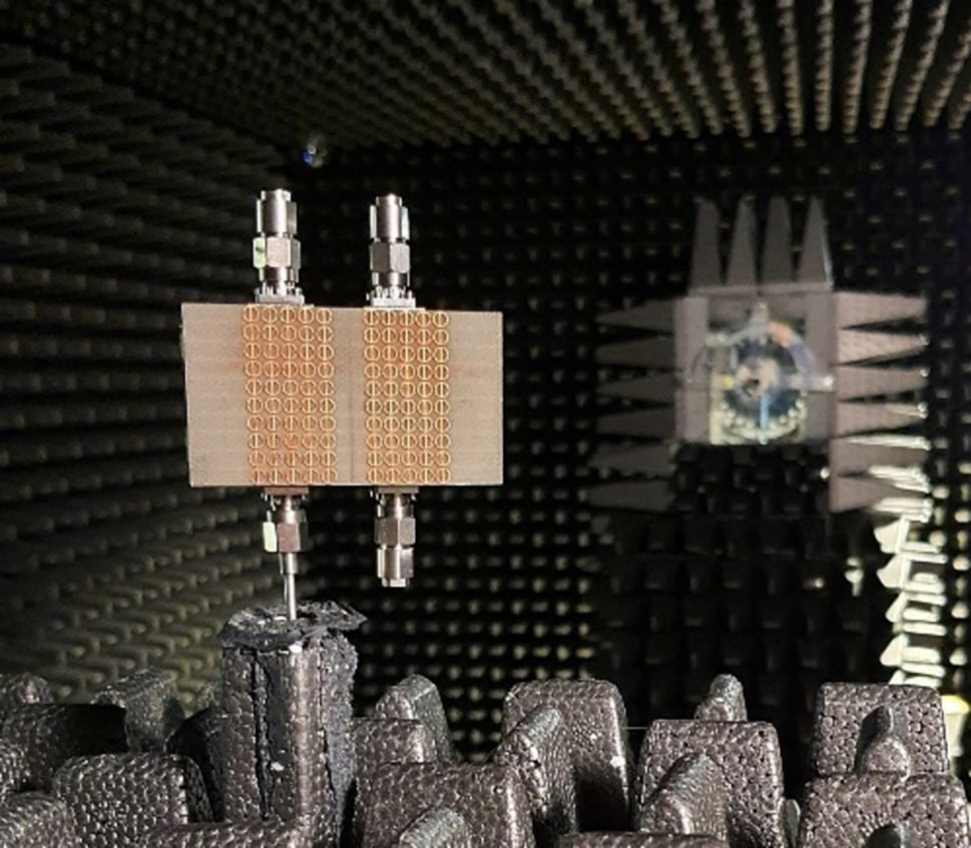
Measurement setup for 2 × 2 antenna array system with N-MTM inside the anechoic chamber.

**Figure 16:**
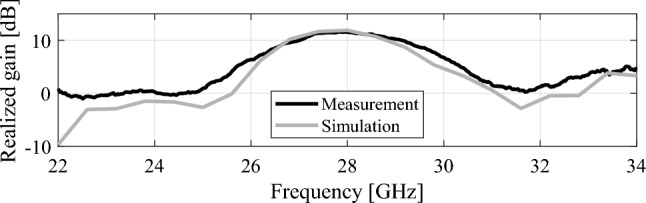
Broadside realized gain versus frequency of the 2 × 2 antenna array system with N-MTM.

**Figure 17:**
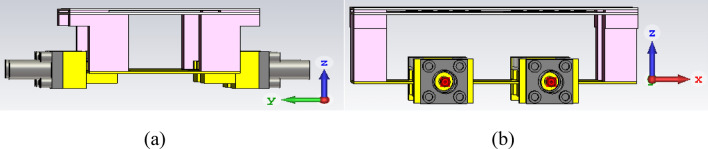
CST Simulation Setup, (**a**) 2 × 2 antenna array system with N-MTM H—Plane (YOZ), (**b**) 2 × 2 antenna array system with N-MTM E—Plane (XOZ).

**Figure 18:**
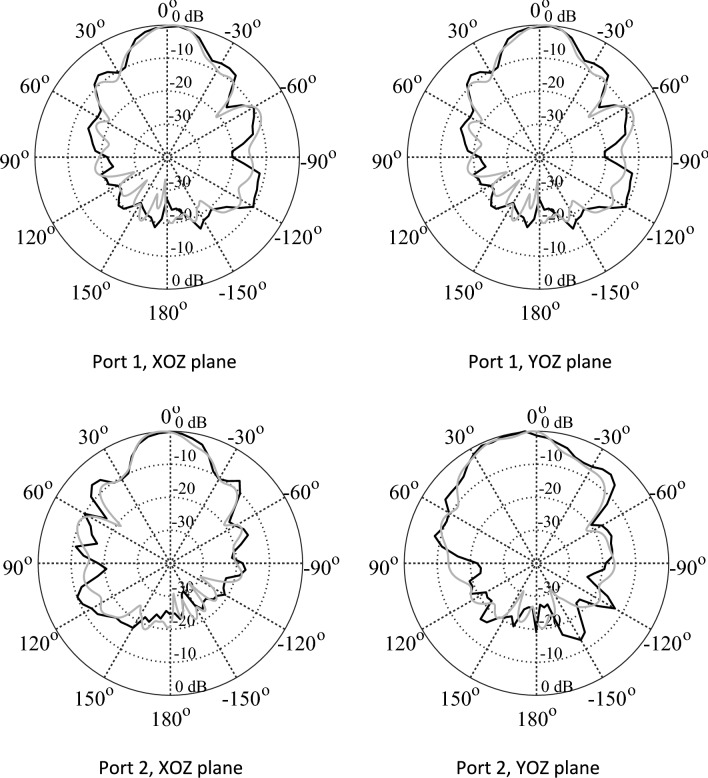
Radiation patterns at 28 GHz: simulation (gray) versus measurement (black).

In Fig. 15, the experimental setup of the proposed 2 × 2 antenna array with double metamaterial layer inside the anechoic chamber is depicted. The design of the four-port antenna system was intended to illustrate the concept of future millimeter-wave array systems, where multiple antenna elements are densely packed together on the same substrate. Although the prototype was connected to only two ports within the anechoic chamber for testing purposes, it is important to note that all ports were functional and tested. The realized gain of the proposed array system was measured in the chamber at the desired frequency of 28 GHz, with the peak gain recorded at 13.5 dBi, as shown in Fig. 16. Furthermore, simulations and measurements of the radiation patterns at the E-plane (XOZ) and H-plane (YOZ) were conducted. The CST setup simulation for the E-plane (XOZ) and H-plane (YOZ) is shown in the Fig. 17. Furthermore, Fig. 18 illustrates the polar plots of the simulated and measured radiation patterns at the XOZ and YOZ planes, respectively. Remarkably, a gain enhancement of 7.55 dB in the E-plane and 7.25 dB in the H-plane was achieved at the resonant frequency of 28 GHz, demonstrating a strong agreement between the simulated and measured results.

### Benchmarking

The proposed antenna has been compared to recent state-of-the-art designs reported in the literature, and utilizing superstrate metamaterial unit cells for gain enhancement at millimeter-wave frequencies. The data gathered in Table 2 indicates that the proposed structure exhibits a superior combination of gain performance and small size. In particular, it offers significantly higher gain than antennas featuring similar physical dimensions. At the same time, it is considerable smaller than the benchmark structures of comparable gain. In the context of negative metamaterials, extensive research has indeed been conducted across various fields. However, there remain unexplored avenues, particularly in emerging technologies like 5G/6G communication systems. These metamaterials hold immense promise for applications such as reconfigurable intelligent surfaces and small base stations. By enhancing electromagnetic wave properties—such as gain and beam steering—our novel design showcases negative metamaterial behavior. Notably, it achieves an impressive wide bandwidth of 68.75%. The modifications introduced to the unit cell, including targeted adjustments to gaps in the metallic ring and the incorporation of middle slots, contribute to the realization of a negative refractive index. Our work contributes significantly to advancing the understanding and utilization of negative metamaterials, opening new possibilities for innovative communication applications and beyond.

**Table 2:** Performance comparison between proposed antenna and state-of-the-art designs operating at millimeter wave range.

Refs.	Centre frequency *f*_*c*_ (GHz)	Basic antenna element	Number of superstrates	Percentage bandwidth (%)	Max. gain (dBi)	Superstrate unit cell array size (L × W)	Antenna efficiency (%)
^23^	28	Slotted patch	1	17	12.7	1.25λ × 1.3λ	95
^14^	29.5	Dipole	3	22.5	10.5	1.18λ × 0.88λ	93
^24^	28	Slotted patch	1	40	15	2.6λ × 3.1λ	N.A
^25^	30	Dielectric patch	1	15.3	15.4	2.9λ × 2.9λ	90
^26^	29.5	Yagi	1	17.2	11.9	13.7λ × 13.7λ	94
^27^	10.5	Microstrip patch	3	42	11.64	1.73λ × 1.73λ	N.A
This work	28	Microstrip patch	2	68.75	13.5	1.59λ × 1.59λ	90

## Conclusion

This paper introduced a novel negative refractive index metamaterial (NIM) designed to enhance the gain of a traditional MPA at a millimeter-wave frequency of 28 GHz. The NIM, with negative effective permittivity and permeability, is employed as a superstrate in two configurations: over a single MPA and over a 2 × 2 array of MPAs. The results demonstrate a significant gain increase of 7.9 dBi when utilizing the proposed NIM superstrate over a single antenna. The area of the 5 × 5-unit cell array with single MPA is compact about 1.59*λ* × 1.59*λ*. The overall MPA antenna array system with unit cell array superior performance with a total size of 5.71*λ* × 3.18λ × 1.5*λ*. The maximum gain for the antenna array is measured at 13.5 dBi, with notable enhancements in both E-Plane and H-plane at the resonant frequency of 28 GHz due to the presence of the superstrate. The proposed NIM antenna design holds promise for diverse applications, including 5G millimeter-wave access points, reconfigurable intelligent surfaces, imaging sensors, and therapeutic devices. By enhancing antenna directivity, we aim to optimize signal transmission and reception in cellular base stations, contributing to improved network efficiency and user experience.

## Data Availability

The datasets generated during and/or analysed during the current study are available from the corresponding author on reasonable request.
